# From cells to cars: Developing an electrochemical techniques and diagnostics course for new battery engineers

**DOI:** 10.1016/j.isci.2024.109739

**Published:** 2024-06-03

**Authors:** Wesley Chang, Matthieu Dubarry, Jeffrey S. Lowe, Shannon Reed

**Affiliations:** 1 Department of Mechanical Engineering and Mechanics, Drexel University, Philadelphia, PA 19104, USA; 2 Hawaii Natural Energy Institute, University of Hawaii at Manoa, Honolulu, HI 96822, USA; 3 Global Virtual Electrification and Powertrain, General Motors, Warren, MI 48092, USA; 4 The Electrochemical Society, Pennington, NJ 08534, USA

## Abstract

We are a team of three battery researchers and engineers who are working with The Electrochemical Society to develop an “electrochemical techniques and diagnostics for batteries” curriculum, comprised of an online course and an in-person workshop. With a combined 40+ years of experience working in battery research and engineering, ranging from academia to electric vehicle manufacturing, we have noticed that there exists a gap in applied electrochemistry knowledge needed to train the rapidly expanding workforce of battery engineers and scientists. In this backstory, we explain the origin story of our team, our motivations for developing the course and the things we have learned in working together. We share our insights into the emerging electric vehicles business and why we believe electrochemistry education will shape the future of this industry.

**Figure:**
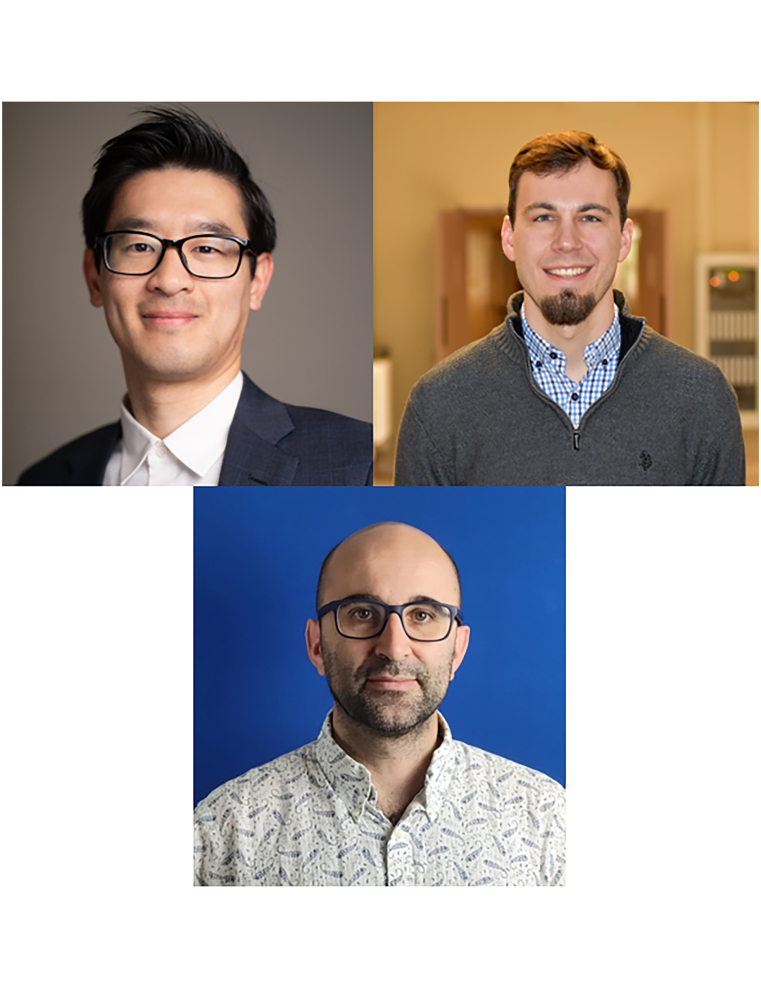
Above image: The instructors of the The Electrochemical Society’s Electrochemical Techniques and Diagnostics Course are depicted (top left: Wesley Chang, top right: Jeffrey S. Lowe, bottom center: Matthieu Dubarry).

We have noticed that there exists a gap in applied electrochemistry knowledge needed to train the rapidly expanding workforce of battery engineers and scientists.

Looking at the main topic and issues from different angles really helped finding the right angle to approach a problem to make it understandable to a wider audience.

We are striving to create content that allows this audience to successfully find a job in the battery or EV industry, and to start their jobs on day one ready to conduct battery diagnostics.

I think we all wanted to do this because each one of us has an interest in not just doing the research but also disseminating the knowledge that is built within individual research labs over many years, to the broader community.

As an industry professional, I have found that collaborations with academic researchers have allowed us to bring cutting-edge research into the development of new products. In turn, I hope that our work in industry provides a practical application of the research being conducted by academics.

We hope that our content finds use for many, many people around the world, specifically undergraduates and graduates in fields ranging from electrochemistry to organic chemistry, to mechanical engineering, to materials science and physics, who want to join the emerging battery and EV industries.

## Context and motivation

### What was the motivation for launching this course and how did you come to work together?

#### The importance of battery education

Roughly 30% of greenhouse gas emissions come from the transportation sector, primarily driven by combustion vehicles that have dominated this market for the past century. The rechargeable Li-ion battery has played a key role in shifting this industry to electric vehicles, with the inventors recently receiving the 2019 Nobel Prize. In 2024, we are witnessing substantial global efforts to scale up Li-ion battery production; from 400 GWh per year to many TWh per year. This means the world will be making more batteries in year 2030 than in the entire history of the battery manufacturing industry. Li-ion batteries are no longer a question of science—they are a question of scale. How quickly and effectively can we train a large enough workforce to develop these battery gigafactories all around the world? Battery production for electric vehicles is a complex process with minimal margins for error. It is imperative that accurate and robust diagnostics of battery health, either during manufacturing or under operation, are carried out to ensure consumer safety. Yet, with minimal formal training for battery or electrochemical engineering in traditional university engineering curricula, this presents a lack in workforce development that needs to be met. We believe this current workforce development initiative can help to close this gap.

#### Training new battery scientists

**Wes Chang:** I heard about this initiative when The Electrochemical Society (ECS) first released it back in December 2022. Having worked on a mixture of fundamental academic projects as well as applied research directly with automobile companies such as General Motors and Mercedes-Benz during my PhD, I saw a clear gap that often exists between the research done in a lab and the practical needs of EV operation. To me, this initiative seemed like an excellent way to close those connections and I was very interested in being a part of it, but felt that I needed a team to truly put out a good product. I thought back to my work with General Motors and remembered a teammate, Jeff Lowe, who I know had previous experience with teaching electrochemistry and was very interested in mentoring incoming engineers at GM. Naturally, I reached out to Jeff about this and he expressed immediate interest. At the same time, I heard from Matthieu in a Slack channel for battery scientists, who posted about finding a team for this. I had known of Matthieu from his many years of work in the field of battery diagnostics, though we had never interacted before. I reached out, we set up a first team meeting, spent a few months working on the proposal, and were very excited when we eventually were tasked by the ECS to carry this out!

**Matthieu Dubarry:** In the last five years or so, and at least for my little niche of knowledge, I have noticed that literature has become more and more a minefield with accepted errors (cathode/anode instead of the positive and negative electrodes, OCV in lieu of low-rate charge or discharge …) and many publications lacking the theoretical background to properly use the techniques I have been working on. While some bad studies will unfortunately always slip through the cracks, the problem was that some were being used as a reference by newcomers to the field because of the lack of recent authoritative open-access work. This motivated me to author and co-author a string of open-access reviews and perspective articles on battery testing, diagnosis, and state of health. While these papers were well received, the information was still too dispersed. The Electrochemical Society proposal for a course devoted to electrochemical techniques and diagnostics for batteries came at the perfect time to provide a unique opportunity to integrate everything in a comprehensive and hopefully authoritative package. Since my knowledge is limited to a small part of the field, I reached out on a Slack battery modeling channel to see if anyone with complementary expertise would be interested in teaming up and that is how I met Wes and Jeff.

**Jeff Lowe:** For me, teaching is one of the most rewarding professional endeavors. One of my teaching mentors said that teaching is how you live forever since you pass along what you’ve learned to your students. By teaching this course we are passing along knowledge to our students. Though, perhaps more importantly, we also have the platform to pass along wisdom beyond the content of a traditional electrochemistry curriculum. For example, I worked with a local Chevrolet dealership to create a video showcasing one of GM’s newest electric vehicles, the 2024 Chevy Blazer EV. Using the vehicle, I was able to demonstrate where we’re at today in the commercialization of batteries for EVs. This experience was only possible through the partnership with ECS, and it’s something that isn’t normally conceivable in the context of a traditional curriculum.

### Challenges and benefits

#### Did you encounter any challenges or benefits of working on a team composed of people from different backgrounds and expertise? How did you bridge the gap among different disciplines and global locations? How do you prepare to teach, interact, and solve conflicts in interdisciplinary settings?

**Wes Chang:** I think the main challenge for us has been the realization that developing a whole curriculum on battery diagnostics is not a quick task. Developing original content that is at the same time directly applicable to our audience (undergraduate or graduate engineers in a field tangential to batteries, who want to join the battery and EV workforce) requires a lot of time to review every single concept. Our slides have to provide information that is easily digestible, yet covers all possible facets of battery diagnostics. The figures we use should be from the most credible sources. Our video recordings have to be edited and re-edited. We are constantly communicating back and forth to make sure our individual content is integrated with each other in one coherent series.

**Matthieu Dubarry:** As Wes mentioned, the main challenge comes from the enormity of the task and the level of quality that needs to be maintained throughout, even on topics we are less familiar with. The recording of a professional eLearning class was also completely new for me, but I am sure the end result will be orders of magnitude better than a traditional pre-recorded slide presentation! I really enjoyed working with Wes and Jeff who have a different background. Looking at the main topic and issues from different angles really helped finding the right angle to approach a problem to make it understandable to a wider audience.

**Jeff Lowe:** I think Wes and Matthieu hit the nail on the head with this one. Developing good, practical content takes time, and that has been the largest challenge for me. Though, on that same note, I believe that working in a team has allowed us to generate content that’s more diverse and more impactful. I’ve benefited greatly from working with Wes and Matthieu since they both have a strong academic background in electrochemistry. In fact, although our collaboration has been completely virtual at this point, I don’t feel like this has hindered us. Virtual collaboration tools have come a long way in the last several years. We have relied on internet browser-based applications for slide editing, cross-platform messaging applications for day-to-day updates, and video chats for bi-weekly communication. Moving forward, I expect that we’ll see an expansion of these types of virtual collaborations, which will be a great benefit to science!

### Approach

#### Describe your approach to launching this course

**All:** We are thankful to have a wonderful support team and consulting team (ThinkLearnEngage, LLC) setup by The Electrochemical Society, who have helped with project management, video recording and editing, marketing, and much more. This effort cannot be done by a single individual—it requires many people working closely together to develop the best educational product that is not only technically useful but also visually appealing and user-friendly. For the content, we made sure to seek the most trustworthy sources and ensure proper citations throughout the course. We know we do not have the time to go deep on the many topics to cover, therefore it is essential to provide enough links to guide students toward the best possible paper on the topic.

### Interdisciplinary collaboration

#### How did the decision to branch out from research come about?

**Wes Chang:** I think we all wanted to do this because each one of us has an interest in not only just doing the research but also disseminating the knowledge that is built within individual research labs over many years, to the broader community. So, a lot of this for us is driven by personal interest to see some of the more nuanced understandings of battery diagnostics be brought to light.

**Matthieu Dubarry:** I agree with Wes. The primary goal is outreach and to give back to the community all of what we learned in previous years.

### Communication

#### When launching this course, who is your targeted audience and how will you reach them?

**All:** Our target audience is graduate (MS and PhD) students, as well as undergraduates or current industry engineers who have an interest in joining the battery or EV industries. The audience could be engineers who have had some prior experience with electrochemistry but not batteries, or other scientists in fields ranging from organic chemistry to physics who would like to enter the battery engineering market. We are striving to create content that allows this audience to successfully find a job in the battery or EV industry, and to start their jobs on day one ready to conduct battery diagnostics. Of course, there are some things that one learns on the job through hands-on usage of battery hardware and software, but it helps a lot if they are shown the most immediate and relevant tools and concepts beforehand.

### Future

#### What are the future outlooks of the course? What tips would you give to anyone considering undertaking this type of interdisciplinary work?

**Wes Chang:** As we complete the online portion of the course, we will move toward organizing an in-person workshop. We think this in-person workshop of a number of selected participants will be very useful, as we’ll have people work through demos on battery cyclers and potentiostats, as well as provide a networking opportunity for them to meet each other, to meet battery research faculty, and to meet industry representatives.

We hope that our content finds use for many, many people around the world, specifically undergraduates and graduates in fields ranging from electrochemistry to organic chemistry, to mechanical engineering, to materials science and physics, who want to join the emerging battery and EV industries. Importantly, we hope that we clarify common misunderstandings of battery diagnostics methods and tools.

**Jeff Lowe:** I would encourage everyone to seek out interdisciplinary collaborations in their work. Even though they may not fulfill their primary job function, in the long run, these collaborations are extremely fruitful. As an industry professional, I have found that collaborations with academic researchers have allowed us to bring cutting-edge research into the development of new products. In turn, I hope that our work in industry provides a practical application of the research being conducted by academics.

#### Anything else you’d like to share?

A listing of our course can be found here: https://www.electrochem.org/batteries-techniques-diagnostics/

You can learn more about The Electrochemical Society’s Battery Workforce Development Project by reaching out to Shannon Reed, Director of Community Engagement – Shannon.Reed@electrochem.org.

### About ECS

#### The Electrochemistry Society (ECS)

The nonprofit professional Electrochemical Society has led the world in electrochemistry and solid state science and technology and allied subjects since 1902. We advance scientific theory and practice through publications, meetings, continuing education, and collaboration. Our robust global membership develops solutions to the planet’s major challenges. Scientists, engineers, and industry leaders share research at ECS biannual, co-hosted, and sponsored meetings. The ECS Digital Library on IOPscience hosts abstracts and highly peer-reviewed articles from publications including *Journal of The Electrochemical Society* (the oldest journal in its field), *ECS Journal of Solid State Science and Technology*, and new open-access *ECS Sensors Plus* and *ECS Advances*.

